# Diagnostic Performance of Kato Katz Technique and Point-of-Care Circulating Cathodic Antigen Rapid Test in Diagnosing *Schistosoma mansoni* Infection in HIV-1 Co-Infected Adults on the Shoreline of Lake Victoria, Tanzania

**DOI:** 10.3390/tropicalmed3020054

**Published:** 2018-05-29

**Authors:** Humphrey D. Mazigo, Jorg Heukelbach

**Affiliations:** 1 Department of Medical Parasitology, School of Medicine, Catholic University of Health and Allied Sciences, P.O. Box 1464, Mwanza, Tanzania; 2 Department of Community Health, School of Medicine, Federal University of Ceará, Fortaleza CE 60430-140, Brazil; heukelbach@web.de; 3 College of Public Health, Medical and Veterinary Sciences, Division of Tropical Health and Medicine, James Cook University, Townsville 4811, Queensland, Australia

**Keywords:** *Schistosoma mansoni*, HIV-1, point-of-care circulating cathodic antigen, sensitivity, specificity, adult, Tanzania

## Abstract

Background: The diagnostic performance of the Kato Katz (KK) technique and the point-of-care circulating cathodic antigen (POC-CCA) test in detecting *S. mansoni* infection in the presence of the human immunodeficiency virus-1 (HIV-1) infection has remained inconclusive. The present cross-sectional survey compared the diagnostic performance of the KK technique and the POC-CCA test in diagnosing *S. mansoni* infection in an adult population co-infected with HIV-1 in northwestern Tanzania. Methods: Single urine and stool samples from 979 adults were screened for *S. mansoni* infection using both the KK technique and POC-CCA tests. To compare the performance of the two diagnostic tests a combined artificial gold standard was created, based on either an egg-positive KK technique or a POC-CCA-positive test. Results: Based on the KK technique, the prevalence of *S. mansoni* was 47.3% (463/979, 95% CI: 44.2–50.4), as compared to 60.5% by the POC-CCA test (592/979; 95% CI: 57.4–63.5). The overall sensitivity and specificity of the POC-CCA test were 92.5% (95% CI: 89.4–94.9) and 73.3% (95% CI: 69.6–76.8), respectively. In the HIV-1 seropositive group, the sensitivity and specificity of the POC-CCA test were 78.1% (95% CI: 60.0–90.7) and 45.9% (95% CI: 35.8–56.3). Using a combined gold standard, the sensitivity of the POC-CCA test increased to >90% in both subgroups whereas that of the KK technique in the HIV-1 seropositive group was low (49.5%; 95% CI: 39.6–59.5). Conclusion: In the presence of HIV-1 co-infection, the KK technique attained a very low sensitivity. The POC-CCA test offers the best option for the rapid screening of *S. mansoni* infection in communities with a high prevalence of HIV-1 infection.

## 1. Introduction

Intestinal schistosomiasis caused by *Schistosoma mansoni* remains one of the Neglected Tropical Diseases that is highly endemic in communities living along the shorelines of Lake Victoria in northwestern Tanzania [1,2]. The disease affects individuals of all age groups in these communities, with extremely high infection intensity [3,4,5]. 

Traditionally, the estimation of prevalence and intensity of *S. mansoni* infection in endemic areas is done using the Kato Katz (KK) technique [6]. The KK technique is regarded as a gold standard for diagnosis [6]. In addition, the KK technique provides quantitative information on egg intensity as an indicator of host helminth burden [6]. The technique is highly sensitive in areas with high endemicity levels, but less sensitive in areas where communities have light to moderate intensity of infection [7]. KK technique sensitivity can be increased by repeating stool samples to be collected and tested over consecutive days [7]. This adds more cost to the control programs and may also result in the withdrawal of study participants [7]. Moreover, the use of this technique for post-praziquantel treatment monitoring can lead to an overestimation of the effects of treatment [7].

In the past two decades, human immunodeficiency virus-1 (HIV-1) infection has been recognized as an important confounding factor in the parasitological diagnosis of *S. mansoni* infection using the KK technique [8,9,10,11]. In HIV-1/*S. mansoni* co-infected individuals, the excretion efficiency of *S. mansoni* eggs is reduced, with more eggs retained in the body [8,12]. Excretion of *S. mansoni* eggs from an infected individual is a function of the immune system determined by CD4^+^ Th_2_ type levels [11]. HIV-1 kills CD4^+^ Th_2_ cells, which may affect the excretion efficiencies of *S. mansoni* eggs; this in turn affects the sensitivity of the KK technique [11]. One study reported reduced *S. mansoni* egg excretion in HIV/*S. mansoni* co-infected individuals in western Kenya [11]. This has significant implications on the parasitological diagnosis of *S. mansoni* infection using the KK technique [11,13], especially in adult individuals likely to be co-infected with HIV-1 [10]. This can lead to a misclassification or missed diagnosis in individuals with light to moderate intensity. In addition, individuals co-infected with HIV-1 infection are likely to retain *S. mansoni* eggs in their bodies, which can lead to development of severe hepatosplenic morbidities [14]. 

To overcome these challenges, a point-of-care circulating cathodic antigen (POC-CCA) test that detects *S. mansoni* antigen released by live adult worms in the urine samples of infected individuals has been developed [15,16]. The test has been widely scrutinized [17] and has shown to have a higher sensitivity than the KK technique [7,16,17,18,19,20,21]. A single POC-CCA test has been shown to be more sensitive than six KK thick smears [7]. However, the performance of the KK technique and POC-CCA test in detecting *S. mansoni* infection in the presence of HIV-1 infection has remained inconclusive. The present survey was designed to compare the performance (sensitivity and specificity) of the KK technique and the POC-CCA test in diagnosing *S. mansoni* infection in the adult population either co-infected or not with HIV-1 in northwestern Tanzania.

## 2. Materials and Methods

### 2.1. Study Area

The study was performed at Sangabuye, Kayenze, Igalagala, and Igombe, four villages located at the southern shore of Lake Victoria, in the Ilemela district (32–34° E and 2–4° S), Mwanza region, northwestern Tanzania [10,22]. These are fishing villages highly endemic for *S. mansoni* infection [4]. A high proportion of adult individuals is co-infected with HIV-1 [10]. In these communities, Lake Victoria is the main source of water for domestic and economic use, such as washing, cooking, and fishing. Mass drug administration of praziquantel is focused on schoolchildren [1].

### 2.2. Study Design, Sample Size, Sampling Procedure, and Inclusion Criteria

This was a nested data analysis based on a cross-sectional study conducted between September and December 2012 [10,14,22]. It included all individuals aged 15–55 years living in the selected villages, with no history of praziquantel use in the past six months and no antiretroviral therapy for those who were diagnosed with HIV-1 infection. Participants were randomly selected from randomly-selected households. The sample included in the study and the selection criteria for the study participants have previously been described by Mazigo et al. (2014) [10]. 

If selected individuals declined to participate or were not present at the household after multiple attempts, a new member of the household was randomly selected. For households that remained vacant after multiple visits, a neighboring household was selected.

### 2.3. Data Collection

#### 2.3.1. Questionnaire

A pre-tested questionnaire was used to collect the participant’s demographic information, history of praziquantel drug use, previous HIV-1 testing, and the outcome and history of the antiretroviral treatment. 

#### 2.3.2. Human Immunodeficiency Virus-1 Screening

Testing for HIV-1 infection followed national guidelines, which require participants to be counselled by a qualified nurse. By the time of this study, the Tanzanian algorithm for HIV testing recommended the use of the qualitative rapid tests, Determine^®^ and UN-Gold^®^ [23]. In all identified HIV-1-positive participants, venous blood samples were obtained for CD4^+^ analysis by FACSCalibur machine.

#### 2.3.3. Parasitological Screening for *Schistosoma mansoni* Infection

A single stool sample was collected in a labeled container from each participant. Four thick KK smears were prepared following standard procedures [6] using a template of 41.7 mg (Vestergaard Frandsen, Lausanne, Switzerland). The prepared KK slides were independently examined after 24 h for the presence of *S. mansoni* eggs, and the eggs were counted and recorded. To avoid inter-observer bias, all the slides were examined by one experienced microscopist. For quality control, 10% of the negative and positive slides were re-examined by a reference technician.

#### 2.3.4. Circulating Cathodic Antigen Test

A single urine sample was collected in a labeled container from each participant and examined for the presence of the circulating cathodic antigen (CCA) using the POC-CCA tests following the manufacturer’s instructions (Rapid Medical Diagnostic- http://www.rapid-diagnostics.com/) [16]. Trace results were considered as positive. All laboratory technicians involved in CCA testing were blinded for the KK parasitological and HIV-1 results of the study participants.

### 2.4. Data Analysis

A Microsoft Excel spreadsheet was used for double data entry, and data analysis was performed using Stata Version 15 (StataCorp, 2017, Stata statistical software: release 15. StataCorp LP, College Station, TX, USA). Numbers and percentages were used to describe categorical variables. A comparison of either proportions or categorical variables was done using chi-squared (χ^2^) or Fisher exact tests, where appropriate. For continuous variables, descriptive statistics were reported as means with standard deviation for normally distributed variables and medians with interquartile ranges (IQR) for variables that were not normally distributed. 

The prevalences of *S. mansoni* infection based on KK technique and POC-CCA test and their 95% confidence intervals (CI) were calculated using binomial regression, controlling for the village of residence. The arithmetic mean of the *S. mansoni* egg counts for each participant was calculated from the counts of four KK thick smears and multiplied by 24 to obtain individual eggs per gram of feces. *S. mansoni* egg counts were logarithmically transformed to check for normality but only non-transformed means are presented. Mean egg counts for *S. mansoni* between sex and age were compared using Student t-test (two groups) or ANOVA (more than two groups). The intensity of infection was categorized according to WHO criteria, with 1–99 eggs per gram of feces (epg), 100–399 epg, ≥400 epg defined as low, moderate, and heavy intensities of infection, respectively [24]. 

Understanding the limitation of the KK technique in diagnosing *S. mansoni* infection [25], we constructed an artificial gold standard based on either the KK technique or POC-CCA positivity [26]. Thus, sensitivity, specificity, positive and negative predictive values of the POC-CCA test were calculated using two gold standards: (1) egg-positive by KK technique (as a reference standard for the diagnosis of *S. mansoni* infection); and (2) combined gold standard (infection-positive by either KK technique or POC-CCA-positivity [26]. The sensitivity of the POC-CCA test was calculated using egg-detection (KK technique) as reference standard for diagnosis of *S. mansoni* infection. Specificity, i.e., the percentage of negative individuals correctly identified as such, was calculated as described in [27] and sensitivity was calculated as a percentage of positive individuals correctly identified by the test [27]. In addition, a positive predictive value (PPV = proportion of positive test results that are truly positive) and negative predictive value (NPV = proportion of negative test results that are truly negative) are presented [27]. 

The sensitivity, specificity, positive and negative predictive values, positive and negative likelihood ratios of the KK and POC-CCA tests using a combined gold standard with their 95% CI were determined using the *diagt* or *diagti* command in Stata 15 (StataCorp, 2017, Stata statistical software: release 15. StataCorp LP, College Station, TX, USA), against the gold standard as described above [27]. The performance agreement between the two diagnostic tests was evaluated using Kappa statistics with their 95% CI for each of the sub-study population (i.e., general population, HIV-1 negative, HIV-1 positive). The Kappa values were interpreted according to previous classifications by Landis and Koch [28].

### 2.5. Ethical Considerations

Ethical approval was sought from the joint Ethical and Review Committee of Bugando Medical Centre and Catholic University of Health and Allied Sciences. The study received further ethical approval from the National Ethical Review Committee, National Institute for Medical Research, Tanzania. The study received further permission from the district administrative and division authorities. Kiswahili translated informed consent forms were used to obtain participants’ consent to participate in the study and assent for those who were aged 15–<18 years. Written informed consent was obtained from guardians/parents of all participants aged 15–<18 years. For illiterate participants, a thumb print was used to sign the consent/assent forms after a clear description of the study objectives was given to them. To maintain confidentiality, all the demographic and other clinical data of the study participants were kept in a closed cabinet and whenever the data were accessed no participant names were disclosed; only the identification number was used to identify a participant. All study participants infected with *S. mansoni* were treated with praziquantel (40 mg/kg) according to WHO guidelines [24]. 

## 3. Results

### 3.1. Characterization of Study Population

A total of 979 study participants aged 15–55 years was included in the study. Of these, 45.8% and 54.2% were male and female, respectively (Table 1). The overall mean age was 33.2 ± 10.7 years. Age categories and sex of the study participants are shown in Table 1. Other characteristics of the study participants have been described in detail previously [10,14].

### 3.2. Prevalence of HIV-1 Infection

The overall prevalence of HIV-1 infection was 13.3% (130/979, 95% CI: 11.3–15.6). Female participants had the higher prevalence, as compared to male participants (16% vs. 10%, *p* < 0.01). The age groups >20 years had the highest HIV-1 seropositivity, as compared to the youngest age groups (*p* < 0.01); 31–40 year-olds had the highest prevalence (16.7%).

### 3.3. Prevalence and Intensity of Schistosoma mansoni Infection

Table 2 shows the prevalence of *S. mansoni* infection in relation to the demographic characteristics of the study participants and HIV-1 serostatus, and by the diagnostic method. Based on the KK technique, the overall prevalence of *S. mansoni* was 47.3% (463/979, 95% CI: 44.2–50.4), as compared to 60.5% by POC-CCA test (592/979; 95% CI: 57.4–63.5). 

In both the KK technique and POC-CCA test, males had a significantly higher prevalence than females (Table 2). Similarly, significant age differences in the prevalence of *S. mansoni* infections were noted, with the youngest age group (15–20 years) having the highest prevalence (Table 2). 

The overall geometrical mean eggs per gram (GMepg) of feces was 173.75 epg (95% CI: 151.5–199.3). Male participants had the highest GMepg as compared to female participants (219.1 GMepg vs. 132.1 GMepg, t = 5.3349, *p* < 0.0001). There was a significant difference in GMepg between age groups (*p* = 0.001), with the youngest age group having the highest GMepg (277.2, 95% CI: 196.4–391.3).

Of the 130 HIV-1-seropositive individuals, 40% (52/130) had detectable *S. mansoni* eggs in their stool samples, whereas 75.4% (98/130) were identified as infected, based on the POC-CCA test (Table 2). On the other side, in the HIV-1-negative population, the prevalences were 48.4% and 58.2%, based on the KK technique and POC-CCA test, respectively. 

### 3.4. Diagnostic Performance of Kato Katz Technique and Point-of-Care Circulating Cathodic Antigen Test

In the general population, among 621 individuals diagnosed positive with either diagnostic method, 158 (25.4%) individuals were missed by the KK technique, whereas only 29 (4.7%) individuals were missed by the POC-CCA technique (Table 3). In the HIV-1-seronegative group, 105 (20.3%) of 516 individuals that tested positive in either test were missed by the KK technique and only 22 (4.3%) by POC-CCA test (Table 3). In the HIV-seropositive group, the relative frequency of missed diagnosis by the KK technique was highest—53/105 (50.5%) individuals were missed by the KK technique, as compared to 7/105 (6.7%) by the POC-CCA test (Table 3). Considering the intensity of infection in Table 2, the POC-CCA test missed 10.8%, 3.2% and 2.8% of individuals who had low, moderate, and heavy intensity of infection.

### 3.5. Sensitivity and Specificity of Point-of-Care Circulating Cathodic Antigen Test

#### 3.5.1. Using Kato Katz as a Gold Standard Technique

The overall sensitivity and specificity of the POC-CCA test using the KK technique as a gold standard technique in the general population were 92.5% and 73.3%, respectively (Table 4). In relation to HV-1 serostatus, for the HIV-1-seronegative group, the sensitivity of POC-CCA test was 93.8% and specificity was 78.7% (Table 4). For the HIV-1-seropositive group, the sensitivity and specificity were 78.1% and 45.9%, respectively (Table 4). There was a significant substantial agreement between the two tests in the general population (Kappa = 0.62, agreement = 80.9%, *p* < 0.001) and in the HIV-1-seronegative group (Kappa = 0.70, agreement = 85%, *p* < 0.001). However, a significant poor agreement between the two tests was observed in the HIV-1-seropositive group (Kappa = 0.16, agreement = 53.9%, *p* < 0.001).

#### 3.5.2. Using Combined Gold Standard

In the general population, the sensitivity of the POC-CCA test using a combined gold standard was 95.3% (95% CI: 93.4–96.9). For the HIV-1-seronegative group, the sensitivity of POC-CCA test was 95.7%. In the HIV-1-seropositive group, the sensitivity was 93.3%. The positive and negative predictive values for the general population and HIV-1-seropositive group are shown in Table 4.

#### 3.5.3. Sensitivity of the Kato Katz Technique Using Combined Gold Standard

The sensitivity of the KK technique using the combined gold standard was 74.6% (95% CI: 70.9–77.9) in the general population, Table 5. In relation to HIV-1 serostatus, for the HIV-1-seronegative and -seropositive groups, the sensitivities were 79.7% and 49.5%. For the HIV-1-infected group, sensitivity was 49.5% (95% CI: 39.6–59.5). The positive and negative predictive values of the KK technique both in the general and HIV-1 seropositive group are shown in Table 5.

## 4. Discussion

The main findings from this study indicate that the POC-CCA test is more sensitive than the parasitological KK technique in detecting *S. mansoni* infection among the general study population, specifically in the HIV-1/*S. mansoni* co-infected population. The sensitivity of the KK technique is relatively low, especially in the HIV-positive subpopulation, and the number of adult individuals detected with *S. mansoni* infection by the POC-CCA test was considerably higher than those detected using four KK thick smears. These results have implications for diagnostic methods to be used in mass screening programs in similar settings, especially among adult individuals. 

Many studies in sub-Saharan Africa have recorded similar findings [17,19,20,21]. The performance of the POC-CCA test is influenced by the baseline intensity of *S. mansoni* infection [7]. The high sensitivity of the test is achieved in areas with a high intensity of infection [7] and in areas with a low intensity of *S. mansoni* infection, low test sensitivity has been reported [29]. In our study, the performance of the POC-CCA test was mainly influenced by the intensity of *S. mansoni* infection, and the majority of individuals with a low intensity of infection were missed by the test. The test missed very few individuals who had a moderate or heavy intensity of infection. Previous studies have demonstrated a positive relationship between the intensity of *S. mansoni* infection and positivity of the POC-CCA test [30,31]. We noted a low specificity of POC-CCA test and this partly is explained by the low sensitivities of the KK technique [7]. 

The use of the KK technique as a gold standard in evaluating the performance of other diagnostic tests has its limitations [6,25]. Thus, we developed an artificial gold standard, as previously described by Midzi et al. (2009) [26], to compare the diagnostic performance of the KK technique and POC-CCA test. Using the combined gold standard, the sensitivity of the POC-CCA test both in general and in the HIV-1 seropositive group improved markedly to >90%. The observed sensitivity was higher than that of the KK technique both in the general population and in the HIV-1-seropositive subgroup. Given the fact that adult individuals in *S. mansoni*-endemic areas are likely to carry a low intensity of infection and co-infections with HIV-1 affect the performance of the parasitological technique [9,11], the use of alternative diagnostic tests such as the POC-CCA test is highly recommended in the adult population. 

Using the combined gold standard, the KK technique recorded very low sensitivity in the HIV-seropositive subpopulation, which was below 50%. Partly, reduced egg excretion efficiencies caused by the HIV-1 infection may explain the poor performance of the KK technique in the co-infected adult individuals [9,10,11,32]. Other authors have recorded a low sensitivity of the KK technique in pre-school children populations likely to have a low intensity of infection [18,29,33]. The performance of the KK technique appears to be influenced by the prevalence and *S. mansoni* intensity of infection or transmission intensity [34]. In areas were *S. mansoni* prevalence exceeds 50%, the performance of the KK technique yields results relatively similar to the POC-CCA test [34]. However, in areas with a lower prevalence of *S. mansoni* infection, the performance of the KK technique decreases whereas that of the POC-CCA test is increased [34]. If control programs adapt selective treatments based on parasitological results, a proportion of individuals would miss the treatment, especially in settings with a high HIV prevalence. This proportion will maintain transmission of the disease in that particular setting. Thus, employment of multiple diagnostic tests is highly recommended in epidemiological surveys to increase the chances of detecting targeted infection, especially in adult individuals likely to be have low infection intensity and to be co-infected with HIV-1. The use of multiple diagnostic tests (use of both tests in screening, or in case of limited funding, POC-CCA for screening, and KK for confirmation of diagnosis) allows a more accurate determination of disease burden when planning intervention programs [35]. However, POC-CCA is more expensive, and the control programs need to consider refunding. The cost of the POC-CCA test is about 5.00 USD under field conditions, whereas the cost for the KK technique is only about 1.50 USD per person. 

In general, the KK technique has remained the diagnostic test of choice for epidemiological surveys aiming at estimating the prevalence and intensity of infection [6,18,29]. However, the sensitivity and specificity of the KK technique has remained a topic of discussion for many years [18,34,36]. Authors have documented its low sensitivity in detecting infection in individuals carrying low intensity of infections, especially young children and adult individuals likely to be co-infected with HIV-1 [18,34,36]. This makes the KK technique less useful in areas with low rates of transmission characterized by populations carrying low intensities of infection [18,34]. Partly, the low sensitivity of the KK technique is explained by the day-to-day variability in eggs output and the heterogeneous distribution of the *S. mansoni* eggs within the fecal samples [25]. 

Our study is subject to limitations. Considering the day-to-day variability of *S. mansoni* egg output [25], the use of a single stool sample to examine for *S. mansoni* infection may have led to an underestimation of the prevalence of *S. mansoni* using the KK technique. In addition, the low number of HIV-1/*S. mansoni*-co-infected individuals may have affected the power in assessing the sensitivity and specificity of the two tests. Considering the absence of a diagnostic standard, we combined both diagnostic tests to create an artificial gold standard. We are aware that this method is limited, as the tests—to some degree—are evaluated against their own results, and thus have taken care in the interpretation of respective study results. False-positive detection results can be considered as low, as the KK technique detects eggs of *S. mansoni,* and the POC-CCA specific antigens. Nevertheless, the data shows that a high number of diagnostic cases are missed by the use of only one method. 

## 5. Conclusions

Considering the better diagnostic performance of POC-CCA, especially regarding the sensitivity in the HIV-positive population, and the high number of schistosomiasis cases missed by the KK technique in this group, POC-CCA should be used in screening programs in communities with a high prevalence of HIV infection. An approach using multiple testing techniques for the detection of schistosomiasis cases is recommended. 

## Figures and Tables

**Table 1:** Demographic characteristics of the study participants from four villages, Ilemela district, north-western Tanzania.

Sex	Age Groups (Years)					Total
	15–20 N (%)	21–30 N (%)	31–40 N (%)	41–50 N (%)	51–55 N (%)	
Female	82 (63.1)	179(54.4)	146(50.7)	86(57.7)	38(45.8)	531(54.2)
Male	48(36.9)	150(45.6)	142(49.3)	63(42.3)	45(54.2)	448(45.8)
Total	130	329	288	149	83	979

**Table 2:** Prevalence of *S.mansoni* infection based on Kato Katz technique and point-of-care circulating cathodic antigen test, stratified by sex, age and HIV-1 infection status.

Variable		N	KK Technique	*p*-Value	POC-CCA Test	*p*-Value
			n (%)		n (%)	
Sex	Female	531	212(39.9)	*p* < 0.01	293(55.2)	*p* < 0.01
	Male	448	251(56.1)		299(66.7)	
Age group (years)	15–20	130	81 (62.3)	*p* < 0.01	99(79.1)	*p* < 0.01
	21–30	329	171(51.9)		217(65.9)	
	31–40	288	122(42.4)		161(55.9)	
	41–50	149	56(37.6)		68(45.6)	
	51–60	83	33(39.8)		47(56.6)	
HIV-1 serostatus	Positive	130	52(40.0)	0.07	98(75.4)	*p* < 0.01
	Negative	849	411(48.4)		494(58.2)	
Intensity of *S. mansoni* infection (epg)	1–99	195	195(42.1)	n/a	174(89.2)	*p* < 0.02
	100–399	126	126(27.2)		122(96.8)	
	≥400	142	142(40.7)		138(97.2)	
Overall		979				

**Table 3:** Diagnostic performance of the point-of-care circulating cathodic antigen and the Kato-Katz technique for the diagnosis of *Schistosoma mansoni* infection in an adult population co-infected or not with HIV-1 infection.

Sub-Population	Diagnostic Test		KK Technique		Total
			Positive	Negative	
General population	POC-CCA	Positive	434	158	592
		Negative	29	358	387
Total			463	516	979
HIV-1-seronegative	POC-CCA	Positive	389	105	494
		Negative	22	333	355
Total			411	438	849
HIV-1-seropositive	POC-CCA	Positive	45	53	98
		Negative	7	25	32
Total			52	78	130

KK-Kato Katz technique, POC-CCA-Point-of-care circulating cathodic antigen test.

**Table 4:** Sensitivity and specificity of point-of-care circulating cathodic antigen test using the Kato Katz technique and a combined gold standard in sub-populations of study participants.

Sub-Population	Sensitivity % (95% CI)	Specificity % (95% CI)	Positive Predictive Values % (95% CI)	Negative Predictive Values % (95% CI)	Kappa Statistics
**Using Kato Katz technique as a gold standard**					
General population	92.5% (89.4–94.9)	73.3% (69.6–76.8)	69.4% (65.2–73.3)	93.7% (91.1–95.8)	0.62
HIV-1-seronegative	93.8% (90.8–96.1)	78.7% (74.9–82.3)	76% (71.7–80.0)	94.6% (92.0–96.6)	0.70
HIV-1-seropositive	78.1% (60.0–90.7)	45.9% (35.8–56.3)	32.1% (21.9–43.6)	86.5% (74.2–94.4)	0.16
**Using combined gold standard**					
In general population	95.3% (93.4–96.9)	100% (99.0–100.0)	100% (99.4–100.0)	92.5% (89.4–94.9)	----
HIV-1-seronegative	95.7% (93.6–97.3)	100% (98.9–100.0)	100% (99.3–100.0)	93.8% (90.8–96.1)	----
HIV-1-seropositive	93.3% (86.7–97.3)	100% (86.3–100.0)	100% (96.3–100.0)	78.1% (60.0–90.7)	----

**Table 5:** Sensitivity and specificity of the Kato Katz technique using a combined gold standard in a sub-population of study participants.

Sub-Population	Sensitivity % (95% CI)	Specificity % (95% CI)	Positive Predictive Values % (95% CI)	Negative Predictive Values % (95% CI)
General population	74.6% (70.9–77.9)	100% (99.0–100.0)	100% (99.2–100.0)	69.4% (65.2–73.3)
HIV-1-seronegative	79.7% (75.9–83.0)	100% (98.9–100.0)	100% (99.1–100.0)	76% (71.7–80.0)
HIV-1-seropositive	49.5% (39.6–59.5)	100% (93.2–100.0)	100% (93.2–100.0)	32.1% (21.9–43.6)
